# The antibody repertoire of autoimmune sensory neuronopathies targets pathways of the innate and adaptive immune system. An autoantigenomic approach

**DOI:** 10.1016/j.jtauto.2025.100277

**Published:** 2025-01-30

**Authors:** Christian P. Moritz, Yannick Tholance, Nadia Boutahar, Coralie Borowczyk, Anne-Emmanuelle Berger, Stéphane Paul, Jean-Christophe Antoine, Jean-Philippe Camdessanché

**Affiliations:** a Synaptopathies and Autoantibodies (SynatAc) Team, Institut NeuroMyoGène, MELIS, INSERM U1314/CNRS UMR 5284, Université Claude Bernard Lyon 1, 43 Boulevard Du 11 Novembre 1918, Villeurbanne, 69622, France; b University Jean Monnet, 10, Rue de Marandière, 42270, Saint-Étienne, France; c Department of Neurology, University Hospital of Saint-Etienne, Avenue Albert Raimond, 42270, Saint-Etienne, France; d Department of Biochemistry, University Hospital of Saint-Etienne, Avenue Albert Raimond, 42270, Saint-Etienne, France; e CIRI – Centre International de Recherche en Infectiologie, Team GIMAP, Univ Lyon, Université Claude Bernard Lyon 1, Inserm, U1111, CNRS, UMR530, CIC 1408 Vaccinology, Saint-Etienne, F42023, France; f Department of Immunology and Biotherapies, University Hospital of Saint-Etienne, Avenue Albert Raimond, 42270, Saint-Étienne, France

**Keywords:** Sensory neuronopathy, Autoantibodies, Autoantigenomics, Protein microarray, Immune system pathways

## Abstract

Sensory neuronopathies (SNN) encompass diverse etiologies, with autoimmunity playing a major role through both cellular and humoral responses. To investigate the humoral autoantibody repertoire in autoimmune SNN, we conducted a retrospective cohort study using large Human Proteome-wide protein microarrays (HuProt 3.1, HuProt 4.0, ProtoArrays). We specifically analyzed immune system pathways targeted within the autoantigen repertoire (the autoantigenome). We included 131 participants: 44 patients with non-paraneoplastic autoimmune SNN (12 with anti-FGFR3 and/or anti-AGO antibodies), 8 with paraneoplastic SNN, and 79 controls. Findings were validated in an independent cohort of 16 SNN patients. Overrepresentation of immune-system-related proteins was assessed using the Reactome database, and serum levels of IFN-γ and IL-6 were measured with the Bio-Plex Pro™ Reagent Kit. Autoimmune SNN sera interact with significantly more immune system proteins than healthy controls (ProtoArrays: 271/863 vs. 14/863, HuProt: 112/1694 vs. 39/1694, both p < 0.0001). Overrepresentation was observed across all major immune sub-pathways, including innate and adaptive immune responses as well as cytokine signaling. Anti-FGFR3-positive SNN patients showed more frequent reactivity to immune system proteins than anti-FGFR3-negative ones. The independent SNN cohort validated the overrepresentation of targeted immune system pathways. Validation with dot blot and ELISA confirmed reactivity to TRIM21 and IL-6 and identified anti-IFN-γ-positive SNN patients. IFN-γ levels correlated weakly with levels of anti-IFN-γ antibodies (Pearson's *r* = 0.22, p = 0.03). We conclude that the antibody repertoire of autoimmune SNN targets pathways of the innate and adaptive immune system, potentially reflecting key disease-related immune pathways and highlighting the systemic role of immune dysregulation in SNN.

## 1 Introduction

Sensory neuronopathies (SNN), also known as sensory ganglionopathies affect the dorsal root ganglia (DRG) and often cause sensory neuron cell death. These disorders involve various mechanisms, including autoimmunity which may concern at least 30% of patients with SNN [1,2]. Well-described features include DRG lymphocyte infiltrates primarily composed of cytotoxic T-cells, overexpression of MHC molecule by neurons and satellite cells, and neuron degeneration [1,3]. This pattern – observed in paraneoplastic SNN, SNN associated with Sjögren syndrome (SjS), and some seemingly idiopathic SNN – suggests a prominent role of cellular immunity [1,3,4]. However, humoral immunity is also implicated, as shown by plasma cells and IgG deposits in the DRG, along with circulating antibodies. These antibodies include anti-Hu or anti-CRMP5 antibodies in paraneoplastic SNN and anti-fibroblast growth factor receptor 3 (FGFR3) or Argonaute (AGO) antibodies in non-paraneoplastic SNN [[5], [6], [7], [8]]. The presence of different antibodies in non-paraneoplastic SNN suggests variations in underlying immunological mechanisms. Given that the humoral immune response is inherently multifaceted, involving numerous antibodies against diverse targets with varied roles, a holistic approach becomes crucial. A systematic investigation, therefore, promises deeper insights into autoimmune SNN. Consequently, techniques such as autoantigenomics, which aims to identify and characterize the entire repertoire of targeted autoantigens*,* have emerged as powerful tools for unraveling antibody response [[9], [10], [11], [12], [13], [14], [15]].

This new, more systematic perspective advances our understanding of autoimmune processes, revealing the complexity of antibody responses. Analyzing antibody repertoires is promising due to their capacity to shape immune responses – either positively, by mitigating diseases, or negatively, by worsening existing conditions.

The availability of arrays encompassing thousands of proteins has demonstrated that different conditions, such as diseases, can exhibit a unique antibody repertoire. Such antibodies can target groups of proteins involved in specific functions or pathways – including those related to the immune system – and potentially modulate them [9,10,16]. Since SNN has not previously been studied in this way, we utilized two different protein arrays to analyze the antibody repertoire directed against involved in immune system pathway proteins in autoimmune SNN. Our cohort included cases with anti-FGFR3 or anti-AGO antibodies, whose roles remain unclear [7,22]. Therefore we sought to delineate the targeted pathways in these cases as well. Ultimately, our objective was to describe and analyze the profiles of antibody-targeted proteins, thereby gaining a deeper understanding of the immunopathology of SNN and its potential subgroups.

## 2 Material and methods

### 2.1 Standard protocol approvals, registrations, and patient consents

This retrospective cohort and observational study utilized sera from human participants. Approval was granted by the ethics committee of the University Hospital of Saint-Etienne, France (IRBN 742021/CHUSTE). The study adhered to the Code of Ethics of the World Medical Association (Declaration of Helsinki). All participants provided written informed consent, and participant privacy rights were maintained. No animal experiments were performed.

### 2.2 Study design, patient selection, description of population, and serum preparation

For the protein array analysis, we retrospectively selected patients and their sera samples to constitute 3 study groups: 1) patients with paraneoplastic SNN, 2) patients with autoimmune SNN without paraneoplastic origin, and 3) a control group of patients with other neuropathies (ONP) and healthy controls (HC). For a validation experiment, we added an independent group of SNN patients with the same characteristics as group 2, that was analyzed with an independent HC cohort.

For SNN patients, the selection criteria were: age ≥18 years and match with diagnostic criteria of SNN [17].

In the first SNN group, we included 8 patients of paraneoplastic origin, characterized by the presence of anti-Hu antibodies (1 female and 7 males, median age 58.5 years) [18]. In the second SNN group, we included 28 autoimmune SNN (14 males and 14 females, median age 55.5 years) based on the presence of at least one of the following criteria: a) presence of an associated autoimmune disease: N = 15 including 8 SjS, 2 systemic lupus erythematosus, 2 undifferentiated connective tissue disease, 1 autoimmune hepatitis, 1 sarcoidosis, and 1 chronic bowel inflammatory disease; b) presence of serum autoantibodies: anti-FGFR3 autoantibodies (N = 8), anti-AGO autoantibodies (N = 4), c) subacute course or asymmetrical distribution of the neuropathy at onset and the absence of familial history, toxic (alcohol, chemotherapy), diabetes, or viral context, suggesting that the disorder was autoimmune (autoimmune-suspected cases, N = 8). The sera of 16 SNN patients for the validation experiment (8 males and 8 females, median age 58.2 years) fulfilled criteria c, while any evidence of an underlying autoimmune context (including the presence of autoantibodies without clear autoimmune diagnostics) was used as an exclusion criterion. Hence these 16 patients were referred to as idiopathic, even though an autoimmune context was suspected given the fulfillment of criteria c. In total, among the 44 SNN, 12 had associated autoimmune diseases without anti-FGFR3 antibodies, 5 had only anti-FGFR3 antibodies, 3 had an autoimmune disease and anti-FGFR3 antibodies, 4 had an autoimmune disease and anti-AGO antibodies, and 24 had none of them.

Controls included 58 patients (37 males and 21 females, median age 64.5 years) with ONP including chronic inflammatory demyelinating polyneuropathy, diabetic neuropathy and sensory or sensory & motor length-dependent axonal neuropathies, and 16 HC (10 males and 6 females, median age 59.5 years), as well as 5 independent HC (3 males and 2 females, median age 66.0 years) for the independent validation cohort, all originating from the blood donation service of the French Blood Establishment in Saint-Étienne, France. Case and control cohorts were roughly age-and sex matched. The sera were prepared and stored as previously described [19].

To measure IL-6 and IFN-γ serum levels and their corresponding autoantibodies (anti-IL-6 and anti-IFN-γ), we used serum samples of 113 patients with SNN fulfilling diagnostic criteria of SNN [17]. All these SNN patients could be considered as autoimmune SNN: 18 had anti-AGO antibodies, 52 anti-FGFR3 antibodies, and 10 an isolated autoimmune disease (mostly SjS) without anti-AGO or FGFR3 antibodies; 33 were classified as autoimmune-suspected cases as described above. All the samples were kept at −80 °C until utilization.

Study sizes were defined by the availability of sera.

### 2.3 Protein microarray

Two commercial array products were used. The distribution of patients and controls tested with each of the arrays are given in Table 1. Each serum was tested in a single array. The first array type, ProtoArray v.5.1™ (ThermoFisher Scientific Carlsbad, California, USA) contained 7661 human predominantly full-length proteins raised in Sf9 insect cells and the second, HuProt 3.1™ (CDI laboratories, Baltimore, USA) contained 15,797 human predominantly full-length proteins raised in yeast. The two arrays share 7292 proteins. For the validation experiment we used HuProt 4.0™ (CDI laboratories, Baltimore, USA), containing 17,105 human predominantly full-length proteins and sharing 7294 proteins with with ProtoArray and 15,054 with HuProt 3.1.

**Table 1:** Distribution of patients and controls between the two commercial array products.

Study group	n patients analyzed with ProtoArray®	n patients analyzed with Huprot 3.1®	n patients analyzed with Huprot 4.0®	Total n
**Autoimmune SNN (non-paraneoplastic)**	16	12	16	**44**
With associated autoimmune disease only	2	6	0	8
With anti-FGFR3 antibodies only	5	0	0	5
With associated autoimmune disease and anti-FGFR3 antibodies	2	1	0	3
With associated autoimmune disease and anti-AGO antibodies	0	4	0	4
Autoimmune-suspected	7	1	0	8
Idiopathic cases	0	0	16	16
**Paraneoplastic SNN with anti-Hu antibodies**	8	0	0	**8**
**Control group**	14	31	34	**79**
ONP	7	22	29	58
HC	7	9	5	21
**Total**	38	43	50	**131**

Each patient was tested on separate arrays.

AGO: Argonaute; FGFR3: fibroblast growth factor receptor 3; HC: Healthy control; n: number; ONP: Other neuropathies; SNN: sensory neuronopathies.

For ProtoArray, according to the manufacturer and as described in Antoine et al. (2015) [6], sera were diluted 1:500. Bound IgGs were revealed with Alexa Fluor 647-labelled goat anti-human IgG antibodies using appropriate laser activation. The slides were scanned, and the images analyzed using ProtoArray Prospector v5.0 software. The Chebyshev inequality p value and the z-score were used to identify serum reactivity specific to the paraneoplastic or non-paraneoplastic SNN patients and not present in controls [6].

For HuProt, the arrays were handled as published previously [9,20]. The HuProt 4.0 series was applied by PepperPrint (Heidelberg, Germany) using the same protocol. To define the significantly targeted antigens to be listed as group-specific antigens, we applied the following criteria: technical signal replicates of signals that are above-noise (1.5 SDs above array mean) must have a CV% < 20 %; antigens must be in ≥1 sample of a given group (patient or control) ≥ 2.5 SDs above the mean of the other two groups [9]; the sample with the strongest signal in the analyzed group must be ≥ 0.5 SDs above the strongest signal of the other two groups.

With the two array types, only proteins specific to one study group (not recognized by the other study groups) were retained as group-specifically targeted antigens for further analysis. As recommended [20], we used for each study group the respectively other groups as a joint reference group to identify the autoantigenomes (cf. column “Comparison study groups” in Table 2).

**Table 2:** Numbers of targeted antigens obtained per study group and protein array.

Study group, ProtoArray	N	Comparison study groups
Paraneoplastic SNN	211	autoimmune SNN, ONP/HC
Autoimmune SNN (non-paraneoplastic, autoimmune-suspected)	2174	paraneoplastic SNN, ONP/HC
Anti-FGFR3-positive SNN	1973	paraneoplastic SNN, anti-FGFR3-negative SNN and ONP/HC
Anti-FGFR3-negative SNN	516	paraneoplastic SNN, anti-FGFR3-positive SNN and ONP/HC
HC	120	paraneoplastic SNN, autoimmune SNN, ONP
ONP	101	HC, paraneoplastic and autoimmune SNN

**Study group, HuProt 3.1**		**Comparison study groups**

Autoimmune SNN (non-paraneoplastic, autoimmune-suspected)	1064	ONP/HC
Anti-FGFR3-negative SNN	1039	anti-FGFR3-positive SNN, ONP/HC
Anti-AGO-positive SNN	129	ONP/HC
Anti-AGO-negative SNN	720	ONP/HC
HC	423	ONP, autoimmune SNN
ONP	1707	autoimmune SNN, HC

**Study group, HuProt 4.0**		**Comparison study groups**

Idiopathic SNN	342	ONP, HC
HC	60	Idiopathic SNN, ONP

AGO: Argonaute; FGFR3: fibroblast growth factor receptor 3; HC: Healthy control; N: Number; ONP: Other neuropathies; SNN: sensory neuronopathies.

As a quality control for the protein arrays, we used the identification by the arrays of well-characterized autoantibodies detected by routine antibody-screening of sera for organ and non-organ specific antibodies. With the ProtoArray, the 8 patient with anti-Hu antibodies reacted with proteins of the ELAV-Hu family spotted on the array [21]. With the HuProt 3.1^TM^, gastritis autoantibody against the plasma membrane protein H+, K + -ATPase in a control neuropathy patient and anti-SSA1 and SSA2 antibodies in patients with SSN and SjS were identified by the arrays.

### 2.4 Reactome pathway analysis via PantherDB

Panther 14.0 online software (http://www.pantherdb.org/) was applied to identify the covered reactome categories as published [9]. In detail, we uploaded our repertoire lists (cf. Table 2) and performed a statistical overrepresentation test using human as organism and “Panther Pathways” as the annotation data set. In a pre-selection, we selected only those Panther categories 1) belonging to the parent category “Immune system”, 2) that contain at least three targeted proteins, 3) whose number of targeted proteins is at least 5x higher for one of the SNN subgroups compared to the HCs.

### 2.5 Validation of autoimmune reactivity against TRIM21 by dot plot experiments

SSA/TRIM21/Ro 52kD antibodies were screened using the “Connectivitis Profil 10 Ag dot” kit provided from Alphadia Diagnostic Products (Ware, Belgium).

Briefly, the strips were incubated with diluted patient sera. Unbound or excess autoantibodies were removed by washing. Upon further incubation into alkaline-phosphatase-conjugated goat autoantibodies against human IgG, the enzyme conjugate bound the antigen-antibody complexes. After a second washing step in order to remove excess conjugate, substrate solution was added. Enzyme activity, if present, led to the development of purple dots on the membrane pads. The intensity of the coloration was directly proportional to the amount of autoantibody present in the sample.

Each sample strip contained a cut-off and a positive control. The Blue Diver instrument (Alifax Spa, Padova, Italy) was used to perform the experiment in total automation. Then, the strips were digitalized using a camera and the dots’ intensity determined by Dr Dot software (Alifax Spa).

### 2.6 Measure of IL-6 and IFN-γ levels and their antibodies in the serum of patients

We tested 113 SNN patients for IL-6, IFN-γ, and 111 of them for the autoantibodies directed against these two cytokines (anti-IL-6 and IFN-γ antibodies). Serum levels of IL-6 and IFN-γ were measured using Bio-Plex Pro^TM^ Reagent Kit III and the following BioRad cytokine kits according to the instructions of the manufacturer: IFN-**γ** (171B5019M), IL-6 (171B5006M).

Anti-cytokines antibodies were measured by ELISA by using our established protocol [22] including the control for serum-specific background noise [19], with minor modifications. In detail, we coated ELISA plates with recombinant human IL-6 (0.3 μg/mL, Sino Biological, reference 10395-HNAE) or human IFN-γ (2 μg/mL) in 0.1 M carbonate/bicarbonate, pH 9.6, without glycerol. For IFN-γ, two different recombinant proteins were used, both with human sequences but originating from two different expression systems: IFN-γ (A), reference 11725-HNAS, from Chinese hamster ovary (CHO) stable cells; IFN-γ (B), reference 11725-HNAE, from *E. coli* cells, both from Sino Biological. Sera were diluted 1:300 and secondary antibody 1:2000. Signals were revealed with 1-StepTM Ultra TMB-ELISA substrate (Thermo Scientific, reference 34029) for 20 min and the reaction stopped with 2M sulfuric acid. Positivity for anti-IL-6 and anti-INF-γ antibody was obtained when the reactivity was superior to 4 standard deviations above the mean reactivity of 77 and 62, respectively, HCs. Z-scores were used to refer to the number of standard deviations above the serum reactivity of controls. Our z-score cut-off of z = 4 represents a probability of less than 0.01% to find such a strong outlier just by chance.

### 2.7 Statistical analysis

As in our previous studies [9,20], we applied z-score statistics to define seropositive sera and targeted proteins instead of comparing mean values of immunoreactivities. Categorical data (e.g., proportions of proteins targeted in a given pathway) were analyzed by the Fisher's exact test. P-value ≤0.05 were considered positive after Benjamini-Hochberg correction at level 0.05. For the validating cohort tested on HuProt 4.0, an independent Benjamini-Hochberg analysis was done. Correlation analyses were performed using Spearman *r* (given the rejected normality test via Kolmogorov-Smirnov test) along with p-values. Statistical analysis was performed with MedCalc Statistical Software version 18.2.1 (MedCalc Software, Ostend, Belgium).

## 3 Results

Two different Human Proteome-wide protein microarrays were utilized to analyze the targeted autoantigenome, focusing particularly on immune system pathways in autoimmune SNN. This analysis aimed to deepen our understanding of the disease's pathology from an immune-centric perspective.

### 3.1 Protein array and panther analysis

Table 2 lists the sizes of the autoantigenomes, representing the number of targeted antigens for each study group on each microarray platform.

The lists of specifically targeted antigens were analyzed for overrepresentation of immune-related pathways using PantherDB. Each antigen in our repertoires was assigned to its corresponding Reactome pathways. Subsequently, we determined the number of targeted antigens per experimental group within each distinct Reactome pathway. For instance, out of the 2038 proteins categorized under the “Immune system” pathway, 863 were present on the ProtoArray 5.1. Of these, 271 were targeted by autoimmune SNN patients, whereas only 14 were targeted by HCs (Table 3). Table 3, Table 4, Table 5, and appendices tables A.1 and A.2 summarize the detailed results of the PantherDB analysis.

**Table 3:**
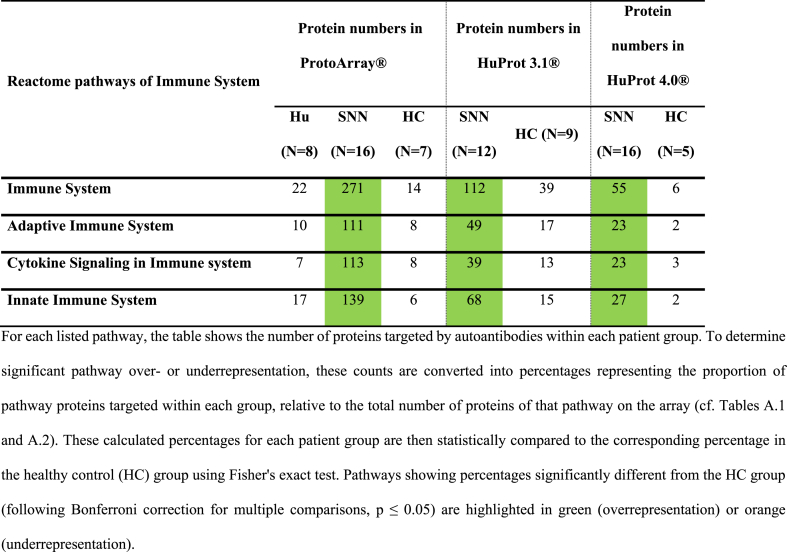
Number of proteins in the immune system pathways recognized by the antibodies of patients with SNN and anti-Hu antibodies (Hu), with other kinds of SNN (SNN) and blood donors (HC) using three array systems.

**Table 4:**
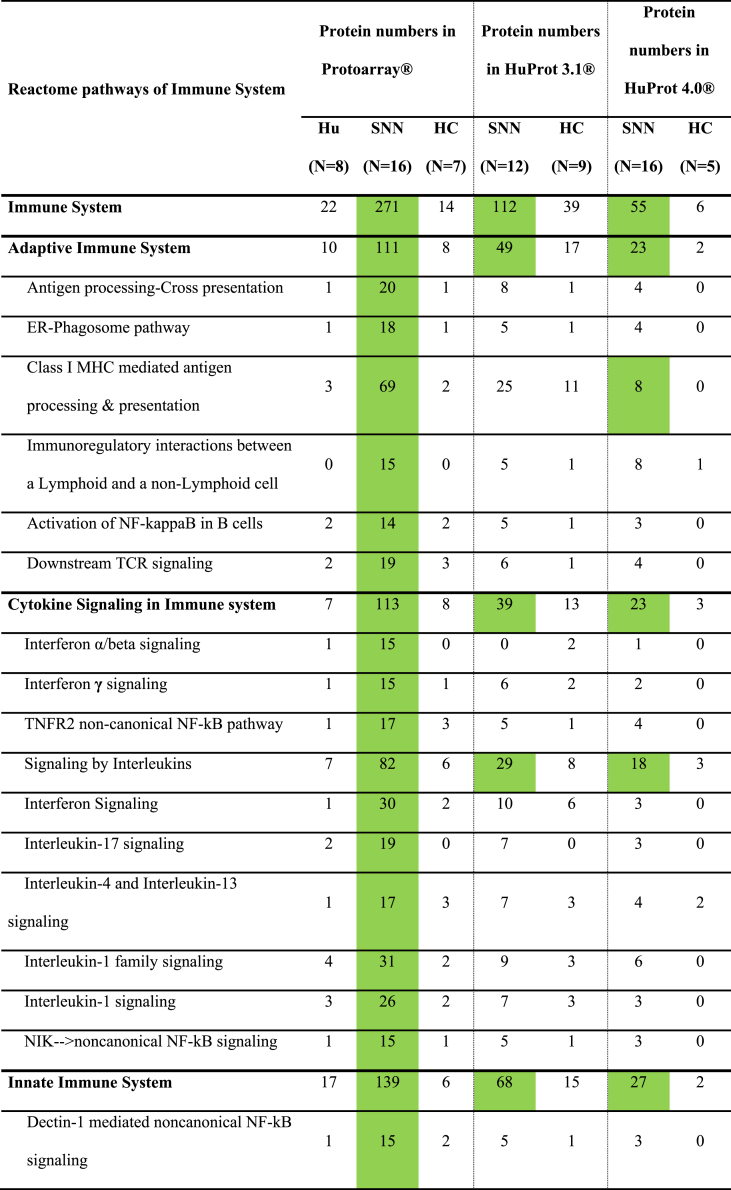

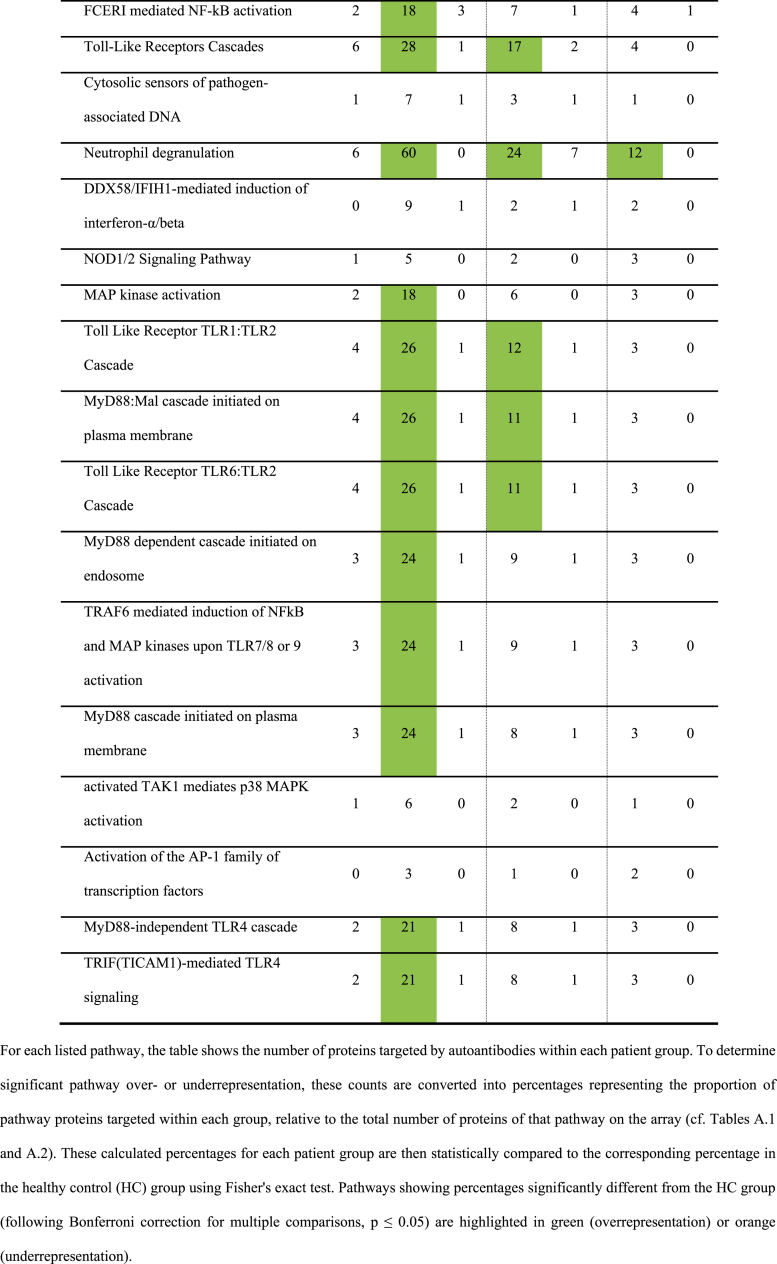
Number of proteins in the immune system pathways recognized by the antibodies of patients with SNN and anti-Hu antibodies (Hu), with other kinds of SNN (SNN), with other peripheral neuropathies (ONP) and blood donors (HC) using three array systems.

**Table 5:**
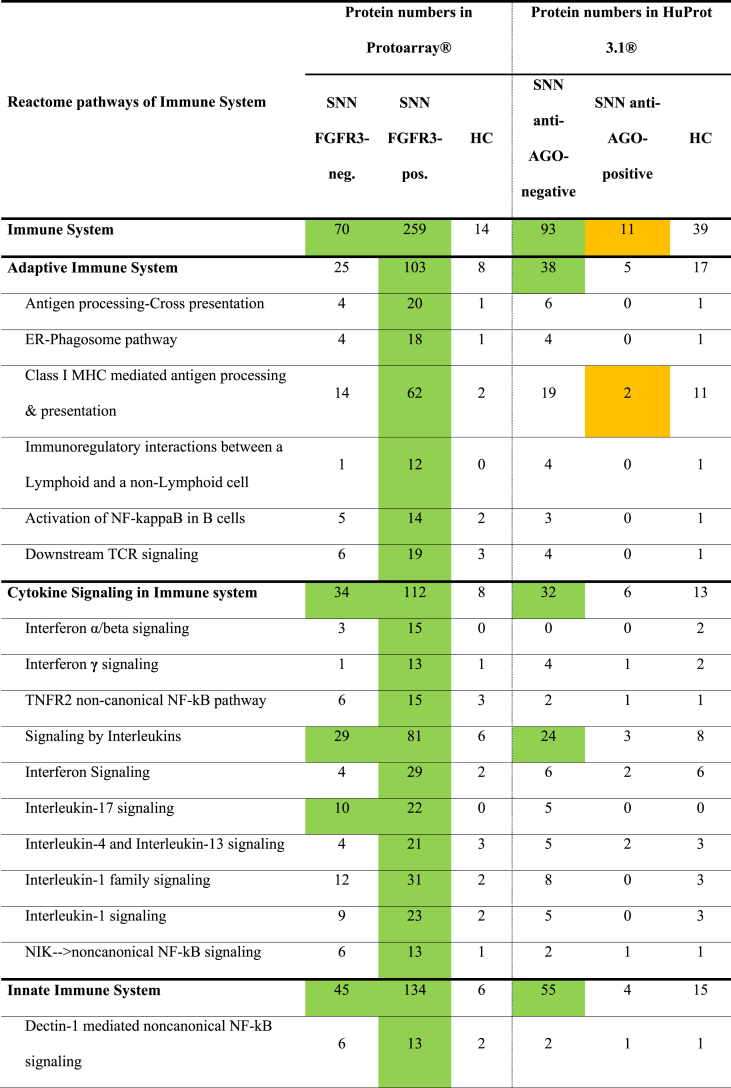

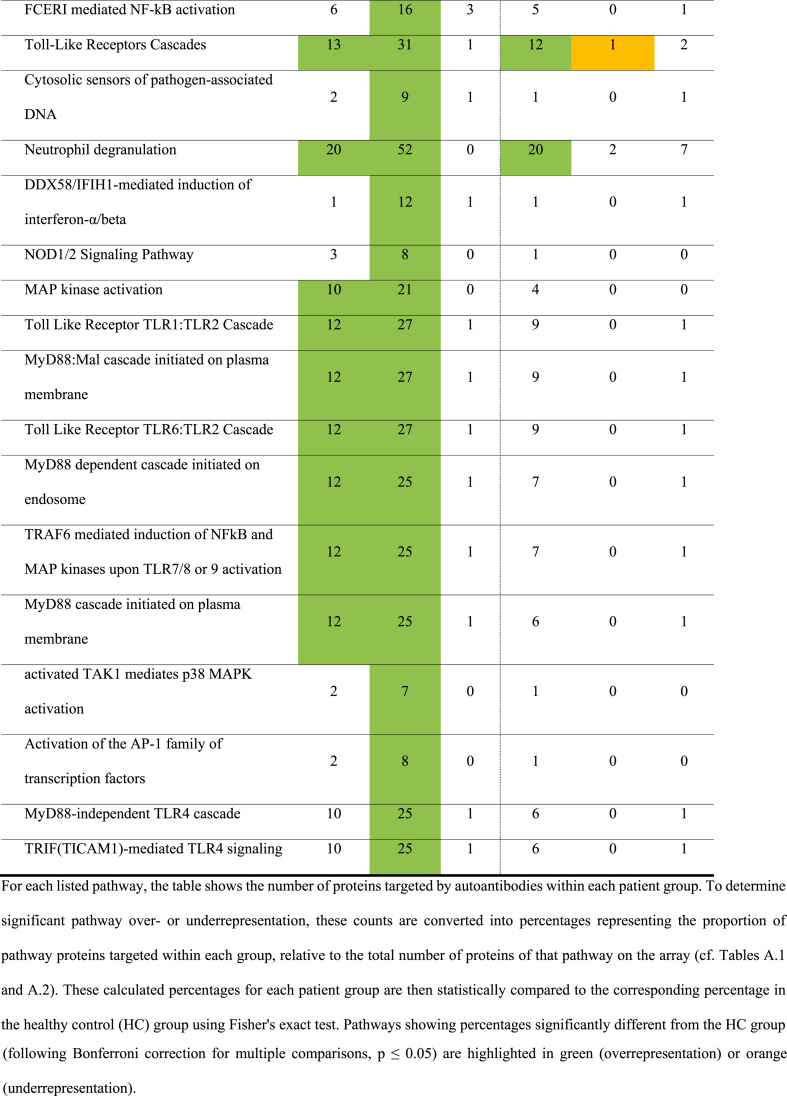
Number of proteins in the immune system pathways recognized by the antibodies of patients with SNN, with and without specific autoautoantibodies (anti-FGFR3 or anti-AGO1 antibodies) using two array systems.

Crucially, the identified proteins exhibited group-specificity, signifying that there was, by definition, no overlap between the protein sets across different study groups.

Autoimmune SNN sera displayed significantly elevated reactivity towards proteins involved in the immune system compared to other groups. Utilizing ProtoArrays, 271 out of 863 (31 %) immune-related proteins were targeted by autoimmune SNN sera, contrasting with 22 out of 863 (3 %) in paraneoplastic SNN (p < 0.0001) and 14 out of 863 [2 %] in HC (p < 0.0001). Similarly, HuProt 3.1 revealed that 112 out of 1694 (7 %) immune-related proteins were targeted by autoimmune SNN sera, compared to 28 out of 863 (3 %) in ONP (p < 0.0001) and 39 out 1694 (2 %) in HC (p < 0.0001; Table 3, appendices tables A.1, A.2).

**Fig. 1:**
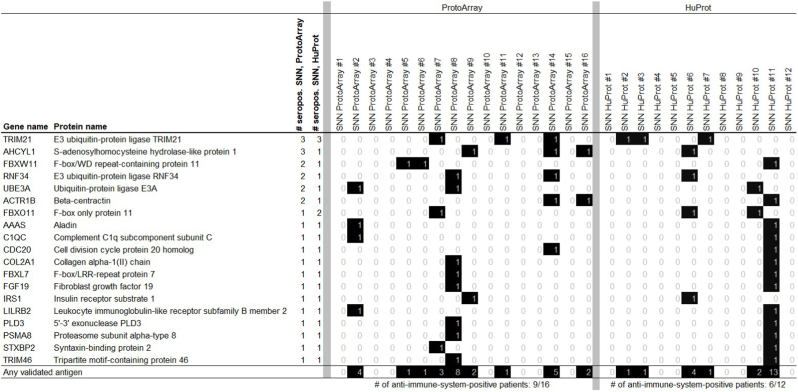
Names and seropositivity pattern of the 19 immune system autoantigens targeted by autoimmune SNN patients and reciprocally validated by both array systems.

Nineteen autoantigens classified as “Immune System” based on Gene Ontology were independantly validated by both array platforms (Fig. 1), indicating robust detection. Among these, E3 ubiquitin-protein ligase TRIM21 (targeted by 3 patients on ProtoArrays and 3 patients on HuProt arrays) and S-adenosylhomocysteine hydrolase-like protein 1 (targeted by 3 patients on ProtoArrays and 1 patient on HuProt arrays) emerged as the most frequently targeted antigens. Remarkably, at least half of the autoimmune SNN patients exhibited seroreactivity against at least one of these 19 reciprocally validated antigens (9/16 on ProtoArrays; 6/12 on HuProt arrays). Fig. 1 visually represents these findings.

Additionally, interleukins and their receptors were targeted by SNN patients on both array types, including IL21 (ProtoArray), and IL-6, IL4R, IL22RA2, IL9R, IL25, and IL7R (HuProt 3.1).

**Fig. 2:**
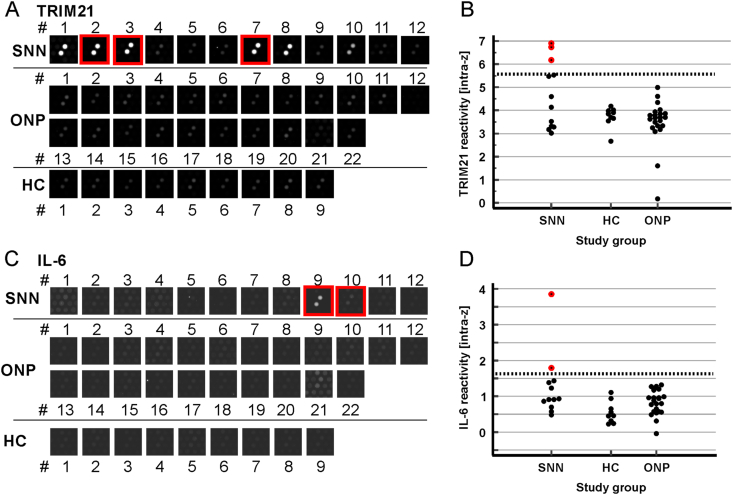
Two representative immune system proteins with their raw data and normalized reactivity plots. A,C: Fluorescence signals depicting the reactivity of 43 human sera (12 SNN, 22 ONP, 9 HC) against TRIM21 (A) and IL-6 (C) protein spots. Sera defined as seropositive in panels B and D are highlighted in red. B,D: Normalized reactivity data (intra-z scores) plotted graphically for all 43 sera across the three study groups. Dotted horizontal lines indicate the positivity threshold (defined as 4 standard deviations above the mean of the ONP + HC group).

Fig. 2 presents representative raw fluorescence signal images of HuProt spots alongside with the corresponding normalized data plots for TRIM21 and IL-6. These two candidates were selected for subsequent validation studies due to high frequency of targeting.

Paraneoplastic SNN did not show overrepresentation of reactions with the “immune system” protein category. They reacted with 3 % (22/863) of proteins on the ProtoArrays, similar to HC (p = 0.99; Table 3, appendices table A.2).

Further analysis of immune system sub-pathways – including innate immune system, adaptative immune system, and cytokine signaling – revealed that autoimmune SNN sera exhibited a greater number of overrepresented pathways (29 out of 34 evaluated pathways) than paraneoplastic neuropathy or HC sera (0 out of 34 for both groups; Table 4). Examples of overrepresented pathways in autoimmune SNN included “Class I MHC mediated antigen processing & presentation” or “Interferon Signaling”.

Using both ProtoArray and HuProt 3.1 platforms, a substantial majority of SNN patients demonstrated reactivity to immune system proteins. On ProtoArrays, 81 % (13/16) of SNN patients targeted at least one such protein, with a median recognition of 4 proteins per patient (interquartile range [IQR]: 35, total range: 0–115). On the HuProt 3.1 arrays, all SNN patients (11/11, 100 %) targeted at least one immune system protein, with a median recognition of 4 proteins per patient (IQR: 9, total range: 1–71). While a similarly high proportion of HC also exhibited reactivity against immune system proteins (ProtoArrays: 7/7, 100 %; HuProt 3.1: 9/9, 100 %) they targeted fewer individual proteins (ProtoArray: median 2, IQR: 1, total range: 1–4; HuProt 3.1: median 5, IQR: 4, total range: 2–10).

Three main categories emerged from the overrepresented pathways identified in the analyses (Table 3, Table 4, Table 5). The first involves the adaptive immune system. Overrepresentation was observed in pathways related to antigen processing and presentation by MHC class I, B cell activation through the NF-kappa B pathway and TCR signaling. These pathways were consistently overrepresented in the autoantigenome of anti-FGFR3-positive SNN.

The second group concerns cytokine pathways and includes signaling by interferon α and **γ** (INFα and **γ**) and interleukin signaling pathways. Among the latter, IL-1, IL-4, IL-13 and IL-17 pathways are overrepresented in the anti-FGFR3-positive SNN autoantigenome.

The last group involved the innate immune system, in particular Toll like receptor (TLR) 1, 2 and 4 cascades and MyD88, the adaptor for inflammatory signaling pathways downstream TLR and IL-1 receptor family pathways. Similar to the adaptive immune system pathways, these innate immune system pathways were overrepresented in the autoantigenome of anti-FGFR3-positive SNN, although some pathways, such as the TLR cascade, were also seen in the autoantigenome of anti-FGFR3-negative SNN.

Notable differences emerged between anti-FGFR3-positive (n = 7) and anti-FGFR3-negative patients (n = 9, Table 5). Although both groups exhibited overrepresentation of immune system targets on ProtoArrays, the extent differed significantly. Anti-FGFR3-positive SNN targeted 259 (30 %) of the 863 immune system proteins (Table 5), whereas anti-FGFR3-negative SNN targeted only 70 of 863 (8 %; p < 0.00001). This trend persists across sub-pathways, with the FGFR3-positive SNN sera demonstrating at least twice the number of targeted proteins compared to negative sera (Table 5).

Similarly, anti-FGFR3-positive SNN sera were involved in many more overrepresented pathways as compared to FGFR3-negative SNN sera: 7 versus 0 in the adaptive immune system, 11 versus 3 in cytokine signaling, 19 versus 10 in the innate immune system (Table 5). These findings suggest that the overrepresentation observed in all SNN is primarily driven by the anti-FGFR3-positive subgroup. Nevertheless, while less pronounced, overrepresentation remains statistically significant in anti-FGFR3-negative SNN (70/863 vs 14/863; p > 0.0001; FDR-controlled ≤0.05). This finding was corroborated using HuProt arrays, confirming the overrepresentation even for anti-FGFR3-negative SNN patients.

In contrast, anti-AGO-positive SNN deviated noticeably from the general overrepresentation pattern observed in other SNN groups. Pathways related to the general immune system, Class I MHC mediated antigen processing & presentation, and TLR Cascades were significantly underrepresented (Table 5). Conversely, anti-AGO-negative SNN mirrored the broader SNN population (i.e., overrepresentation of general immune system, adaptive immune system, cytokine signaling, and innate immune system pathways such as Toll-like receptor cascades).

### 3.2 Validation experiments

#### 3.2.1 Validation of targeted immune system pathways using an idiopathic SNN cohort

To determine whether the observed anti-immune system reactivity in SNN patients could be attributed to underlying systemic immune conditions, we validated our findings in a separate cohort of 16 idiopathic SNN patients lacking any known history of autoimmunity. Using the newest generation of HuProt arrays (version 4.0), we confirmed the significant overrepresentation of central pathways within the autoantigenomes of this independent idiopathic SNN cohort. Compared to an equally independent HC cohort, we observed overrepresentation in “Immune System” as a whole and in major subcategories, including “Adaptive Immune System”, “Cytokine Signaling in Immune System”, and “Innate Immune System” (Table 3). At lower levels of pathways hierarchies, “Class I MHC mediated antigen processing & presentation”, “Signaling by Interleukins”, and “Neutrophil degranulation” were also validated to be significantly targeted by SNN patients (Table 4). Specific examples of significantly SNN-targeted antigens within immune system pathways include E3 ubiquitin-protein ligase TRIM17, interleukin receptor subunits IL13RA2 and IL10RA, and the interferon IFNA16.

### 3.3 Validation of single candidates using independent methods

For independent validation, we selected three candidate targets: TRIM21, IL-6, and IFN-γ.

**TRIM21:** Detected on both array platforms in six SNN patients (three on ProtoArrays and three on HuProt), TRIM21 was prioritized for validation. Of the five patients with available residual serum, all demonstrated anti-TRIM21 reactivity in dot blot experiments (data not shown).

**Fig. 3:**
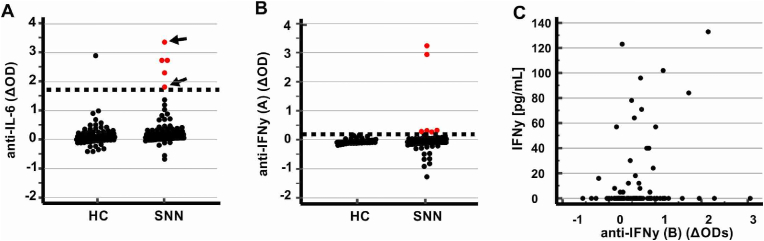
**Immune reactivities against IL-6 and IFN**-**γ antigens in HC and SNN patients.** A: Serological reactivity of HC and SNN patients against IL-6 assessed using independent immunoassays. B: As panel A, but reactivity against IFN-γ (A). C: Correlation plot of IFN-γ levels versus anti-IFN-γ (B) reactivity. Arrows label the two sera that were anti-IL-6-positive on protein arrays. Dotted horizontal lines indicate the threshold for seropositivity (z = 4). Red dots represent SNN patients classified as seropositive.

**IL-6:** Out of 98 SNN patients screened, five tested positive for anti-IL-6 antibodies. Two of these had previously been identified as positive via protein array, thus confirming the initial findings. Among 77 tested HC, we found one anti-IL-6-positive individual (Fig. 3A).

**IFN-γ:** Among 102 SNN sera tested, six were positive for antibodies against IFN-γ (A) (expressed in CHO cells, Fig. 3B) and none for IFN-γ (B) (expressed in *E. coli* cells). None of the 62 HC reacted with IFN-γ (A) or IFN-γ (B).

### 3.4 Targeting of “signaling by interleukins” pathways by SNN

**Fig. 4:**
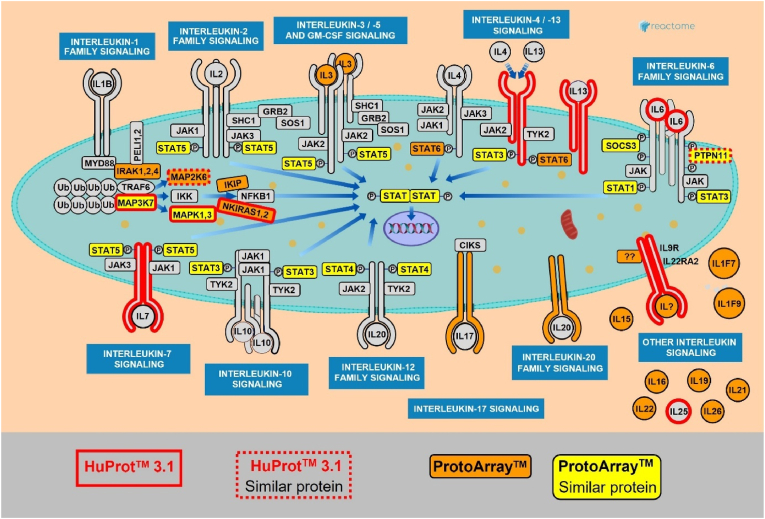
SNN autoantibodies target proteins within the “Signaling by Interleukins” pathways. The figure illustrates key components of interleukin signaling pathways, including interleukins, their receptors, and downstream pathways. Proteins targeted by SNN antibodies are highlighted in red (based on HuProt array data) or orange (ProtoArray). When a closely related protein was targeted instead (e.g., SOCS3 instead of SOCS2), this is indicated by dotted red lines (HuProt) or yellow (ProtoArrays). Note that this figure depicts a selection of pathway components curated from Reactome, supplemented with additional manually chosen elements.

Both anti-FGFR3-positive and negative, as well as anti-AGO-negative SNN cohorts exhibited significant targeting of the “Signaling by interleukins” pathway (Table 5). This overrepresentation was independently validated in anti-FGFR3-negative SNN using a separate cohort and experiment (Table 4). To address the possibility that these findings resulted from concurrent autoimmune conditions, we further validated the results in a third cohort of SNN patients without any known autoimmune background (Table 4). Fig. 4 illustrates the autoimmune targeting of “Signaling by interleukins” pathways.

### 3.5 Correlation analysis of IL-6 and IFN-γ levels with corresponding antibody reactivity

We investigated whether observed antibody patterns targeting cytokine pathways correlated with the circulating levels of these cytokines, potentially reflecting pathway activation. In a cohort of 113 SNN patients, we examined correlations between IL-6 levels and anti-IL-6 antibody reactivity, as well as for IFN-γ levels and anti-IFN-γ reactivity. Furthermore, we compared anti-cytokine antibody reactivities across different SNN subgroups.

Patients with anti-AGO antibodies exhibited significantly higher anti-IL-6 reactivity compared to other SNN patient groups (mean optical density [OD] 0.51 ± 0.59 vs. 0.18 ± 0.43: p = 0.012). Conversely, patients with other autoimmune conditions demonstrated elevated anti-IFN-γ (A) reactivity (mean OD 0.17 ± 0.83 vs. 0.06 ± 0.14, p = 0.03) compared to other SNN patients. No correlation was found between IL-6 level and anti-IL-6 immunoreactivities. However, IFN-γ levels showed a weak but statistically significant positive correlation with anti-IFN-γ (B) reactivity (Spearman *r* = 0.41, p = 0.047, Fig. 3C) when considering IFN-γ values exceeding the detection limit. No significant correlation was observed when analyzing anti-IFN-γ (A) reactivities (data not shown).

## 4 Discussion

Our study addressed a novel autoantigenomic approach to investigate SNN, systematically analyzing immune-related pathways targeted by autoantibodies in SNN patients compared to individuals with other neuropathies and HCs. This comprehensive examination of autoantibody profiles has never been done in SNN and holds promise for advancing our understanding of SNN pathophysiology. By identifying distinctive autoantigenic signatures, we aim to facilitate improved patient classification and ultimately contribute to development of tailored therapeutic interventions [9].

A striking observation emerging from our study is the extensive targeting of proteins involved in both innate and adaptive immunity, as well as cytokine signaling, by the autoantibody repertoire of SNN patients. While recognition of these immune pathway proteins was also evident in HCs and patients with ONP, mostly axonal length-dependent neuropathies, the sheer quantity of targeted proteins was substantially higher in SNN. For instance, using ProtoArray V5 platform, we detected antibodies against 274 distinct immune pathway proteins in SNN patients compared to only 14 in HC cohorts.

To address the possibility that the widespread autoimmune reactivity observed in SNN could result from concurrent autoimmune conditions frequently associated with the disorder, we validated our findings in a control cohort of 16 SNN patients lacking any evidence of underlying autoimmunity. Although the magnitude of anti-immune-system autoimmunity was somewhat reduced in this validation cohort – suggesting a contributing role of coexisting autoimmune conditions in our findings – the overrepresentation of such autoantibodies remained statistically significant. This supports the conclusion that SNN patients possess autoantibodies directed against immune system proteins, independent of any confounding autoimmune condition.

Several lines of evidence support the notion that the observed autoimmune reactivity against immune system proteins is not coincidental. Firstly, our stringent antigen selection process ensured that target proteins in one group (e.g., SNN patients) were not recognized by other groups (e.g., HC or ONP patients). Secondly, IgG antibodies from HCs did not significantly react with any of the immunological pathways targeted by SNN patients. In SNN, up to 50% of proteins within a given immunological pathway were found to be targeted by autoantibodies. Despite variation between the two protein array platforms, consistent identification of overlapping pathways, including “immune system”, “adaptive immune system”, “cytokine signaling”, “signaling by interleukins”, and “innate immune system”, reinforces the validity of the findings. Thirdly, the observed pattern of autoantibody reactivity was not driven by a subset of highly reactive individuals but rather reflected by a broader trend, with 81–100% of SNN patients targeting at least one immune system protein. Finally, this extensive targeting of immune system proteins was absent in paraneoplastic SNN, a distinct subtype of immune-mediated SNN, pointing towards diverging underlying immunological mechanisms within the spectrum of immune-mediated SNN.

Our study revealed another interesting association: SNN harboring anti-FGFR3 autoantibodies displayed a greater number of significantly targeted immune pathways compared to those lacking these antibodies.

Previous research utilizing protein microarrays has demonstrated that the human IgG antibody repertoire encompasses thousands of self-reactive antibodies, organized into intricate networks targeting distinct clusters of highly correlated antigens [23]. These networks, established early in life, initially show considerable overlap among individuals, but evolve over time to become increasingly unique, remaining remarkably stable thereafter. Factors such as age, sex, and the presence of disease exert a profound influence on the abundance and pattern of autoantibodies [24,25], likely reflecting the cumulative impact of an individual's or population's immune experience. In the context of disease, the precise dynamics governing the establishment of this autoantibody profile remains elusive, although it is likely shaped by the specific disease process and persists for years. The antibody repertoire can thus be considered as an indirect reflection of the complex immunological mechanisms underlying disease pathologies and regulation. While the functional roles of these autoantibodies are largely undefined, they may participate in regulatory processes through involvement in idiotypic networks, offer protection against foreign pathogens or malignancy, or contribute to tissue repair and debris clearance.

Recent advancements in autoantigenomics have yielded valuable insights into diverse immune-related diseases.

For instance, a compelling study investigating patients deficient in the autoimmune regulator (AIRE) unveiled a wide range of B cell autoreactivity targeting roughly 100 self-proteins. This breakdown in B cell tolerance exposes a distinctive and personalized repertoire of autoantibody reactivities. Intriguingly, autoantibodies directed against specific cytokines exhibited a strong inverse correlation with type I diabetes, hinting at a potential protective role for naturally occurring human autoantibodies in modulating disease progression [10].

Another investigation employed a multiplex particle-based assay to measure anti-cytokine antibodies, revealing a distinct risk factor for severe COVID-19: the presence of high titers of neutralizing autoantibodies against type I IFN-α2 and IFN-ω. Strikingly absent in asymptomatic or mildly affected cases, these autoantibodies were detected in about 10% of severe COVID-19 cases, providing a biomarker for identifying individuals at heightened risk of adverse outcomes [26].

Our study found a weak but significant positive correlation between IFN-γ levels and anti-IFN-γ (B) antibody reactivity (Fig. 3C), suggesting that the presence of antibodies may indeed mirror the availability of their targeted antigens. This finding supports our hypothesis that the observed antibody patterns partially reflect the underlying physiological state of the system.

Our observation that the patterns of overrepresented pathways differed markedly between anti-AGO-positive and anti-FGFR3-positive SNN patients, compared to the other SNN subtypes, suggests that distinct immune pathways may be implicated in these subgroups. Moreover, these findings hint at potential immunological distinctions between seropositive (either anti-FGFR3 or anti-AGO) and seronegative SNN patients.

A limitation of our study in this respect is the utilization of two different protein array platforms for anti-FGFR3-positive and anti-AGO-positive SNN patients. Due to variations in protein content and representation between the arrays, it is possible that some relevant proteins were overlooked in one of the groups. Nonetheless, because comparison were made between anti-FGFR3/AGO-positive and -negative patients analyzed on the same array type, and since we observed partially contrasting overrepresentation patterns between the two subgroups, we believe these findings are unlikely to be attributable to differences between the arrays.

The identification of distinct subgroups within autoimmune SNN may be helpful to understand disease courses and treatment responses. The hypothesized existence of distinct SNN subgroups is corroborated by the analysis of a SNN cohort specifically examining IL-6, IFN-γ and their respective antibodies. Within this cohort, patients possessing anti-AGO antibodies exhibited elevated levels of anti-IL-6 antibodies compared to other SNN groups. However, these anti-IL-6 antibody levels did not correlate with circulating IL-6 levels. Conversely, in patients with anti-FGFR3 antibodies, circulating IFN-γ levels positively correlated with their corresponding antibody reactivities, suggests a distinct autoimmune mechanism at play.

Finally, in anti-AGO/anti-FGFR3-negative autoimmune SNN patients, the observed correlation between anti-IFN-γ and anti-IL-6 antibodies correlation may reflect yet another divergence in immunological mechanism.

While we observed notable differences in antibody responses between the SNN categories, clear differences in cytokine levels were less apparent. This disparity could arise from blood serving only as an indirect indicator of events within the DRGs. Alternatively, the timing of sample collection – months after disease onset – might have led us to miss transient, early cytokine fluctuations. In contrast, antibody profiles likely reflect more enduring immunological alterations, less susceptible to temporal variations.

Our study is the first to show that SNN antibody repertoire targets components of both the innate and adaptative immune system. While the precise triggers for the emergence of these autoreactive antibodies remain unclear, plausible contributors include exogenous infections, malignancies, or chronic tissue damage, potentially exacerbated by epitope spreading mechanisms [24,[27], [28], [29], [30]]. The stark contrast between paraneoplastic and other forms of autoimmune SNN, despite sharing a common pattern of T-cell mediated pathology, suggests that the immunological mechanisms leading to cytotoxic T-cell activation diverge considerably.

The prevailing consensus recognizes that paraneoplastic SNN arises from cross-reactivity induced by malignant cells expressing an antigen mirroring one found in the nervous system. This shared antigen becomes the primary target of the anti-tumor immune response, inadvertently triggering an attack on neuronal tissues [5].

In non-paraneoplastic SNN, the autoantibody repertoire targets pathways emcompassing INF-α and **γ**, TNF-α, IL1, IL4, and IL17, TLR 1, 2, 4, and 6, MHC I antigen presentation and T-cell receptor signaling. Notably, with the exception of a few variants, these pathways align strikingly with those implicated in numerous autoimmune diseases, most prominently SjS, a condition frequently comorbid with SNN. Early events believed to drive SjS pathogenesis heavily involve the innate immune system [31]. During this initial phase, TLR-3, triggered by RNA strands, plays a predominant role within exocrine glands. Concurrently, INF-α production commences [32]. In SNN, antibody-targeted TLR pathways appear to be activated by cell surface receptors sensitized to heat shock proteins or chromatin proteins [[33], [34], [35]]. Activation of TLR-2 and 4 subsequently stimulates enhanced production of IL-17, a potent proinflammatory cytokine by Th17 lymphocytes [34,36]. Similarly, IFN-**γ**, another crucial player in the early stages of SjS [31], contributes to activating the adaptive immune response. Lastly, the SSN antibody repertoire targeted multiple pathways involving NF kappa B, a transcription factor pivotal in inflammatory processes. Activated by virus, stressors, cytokines, or TLRs, NF kappa B regulates the production of these mediators. It also participates in T-cell activation and has been implicated in numerous inflammatory conditions, including SjS [37].

Furthermore, we identified and validated the presence of Ro52/TRIM21 autoantibodies in five SNN patients. This autoantibody serves as a frequent marker for SjS. Notably, three out of the five SNN patients with confirmed Ro52/TRIM21 antibodies also received a concurrent diagnosis of SjS.

Although our study revealed that autoantibodies target pathways associated with SjS, we addressed the possibility that a co-occurring autoimmune diseases contributed to our findings. While we found evidence supporting their contribution, our results remained consistent even in cases without any known autoimmune background, albeit to a lesser extent. This suggests that our observations extend beyond simply being a consequence of co-existing autoimmune states. Future investigations comparing active immune pathways – perhaps through examining interferon transcriptomic or proteomic signatures – alongside shared autoantibodies, could elucidate the distinct features and similarities between autoimmune SNN and SjS.

Our study does have certain limitations.

1) The relatively small sample sizes within each group, constrained by the expense of protein array technology, may obscure subtle differences. 2) Surface proteins tend to be under-represented on protein microarrays, potentially causing us to overlook relevant transmembrane autoantigens. 3) Our current methodologies allow us to identify reactivities towards immune system proteins but cannot determine the functional effects (such as neutralizing, modulation, or protective) of the antibodies.

## 5 Conclusions

In conclusion, our findings reveal that the autoantigen repertoires in SNN encompass signaling pathways related to cytokines, TLRs, and B and T cell function. For the first time, this study suggests that the antibody profile in patients with non-paraneoplastic SNN reflects underlying immunological pathways likely implicated in disease development. Consequently, the immune pathways contributing to the pathogenesis of SNN may differ between paraneoplastic and other autoimmune forms, as well as between patients with and without anti-FGFR3 antibodies, pointing toward potentially distinct pathogenic mechanisms. These targeted immune pathways may also play a role in the pathogenesis of systemic autoimmune diseases like SjS, offering a plausible explanation for their frequent concurrence. Future research should delve deeper into comparative analysis of immune pathway activity through transcriptomic or proteomic approaches, allowing for direct comparisons among SNN subgroups and with SjS.

## CRediT authorship contribution statement

**Christian P. Moritz:** Writing – review & editing, Writing – original draft, Visualization, Validation, Supervision, Software, Project administration, Methodology, Investigation, Funding acquisition, Formal analysis, Data curation, Conceptualization. **Yannick Tholance:** Writing – review & editing, Validation, Software, Project administration, Investigation, Formal analysis. **Nadia Boutahar:** Writing – review & editing, Software, Methodology, Investigation, Data curation, Conceptualization. **Coralie Borowczyk:** Writing – review & editing, Methodology. **Anne-Emmanuelle Berger:** Writing – review & editing, Methodology, Investigation, Data curation. **Stéphane Paul:** Writing – review & editing, Resources, Funding acquisition, Conceptualization. **Jean-Christophe Antoine:** Writing – original draft, Validation, Supervision, Software, Resources, Project administration, Methodology, Investigation, Funding acquisition, Formal analysis, Data curation, Conceptualization. **Jean-Philippe Camdessanché:** Writing – review & editing, Validation, Supervision, Resources, Project administration, Methodology, Investigation, Funding acquisition, Formal analysis, Data curation, Conceptualization.

## Funding

Parts of the study were funded by the *Association Française contre les Myopathies* (AFM-MyoNeurALP 2), the GROUPAMA Foundation, the 10.13039/501100001659German Research Foundation (DFG; MO 3240/1–1:1), the 10.13039/501100014255Fonds de dotation CSL Behring pour la recherche, and the Centre Hospitalier Universitaire of Saint-Étienne. This work has been developed within the BETPSY project, which is supported by a public grant overseen by the 10.13039/501100001665French National Research Agency (ANR), as part of the second “*Investissements d′Avenir*” program (reference ANR-18-RHUS-0012) and by the FRM (*Fondation pour la Recherche Médicale*) DQ20170336751. The funders had no role in study design, data collection and analysis, decision to publish, or preparation of the manuscript.

## Declaration of competing interest

The authors declare the following financial interests/personal relationships which may be considered as potential competing interests:Jean-Christophe Antoine reports financial support was provided by Fondation pour la Recherche Médicale. Jean-Christophe Antoine reports financial support was provided by AFM-Téléthon. Jean-Philippe Camdessanche reports financial support was provided by 10.13039/501100014255Fonds de dotation CSL Behring pour la recherche. Jean-Philippe Camdessanche reports financial support was provided by Fondation GROUPAMA France. Christian P. Moritz reports financial support and travel were provided by 10.13039/501100001659German Research Foundation. Christian P. Moritz reports a relationship with argenx France SAS that includes: travel reimbursement. Christian P. Moritz reports a relationship with Grifols Inc that includes: funding grants. Jean-Philippe Camdessanche, Jean-Christphe Antoine, Nadia Boutahar has patent #WO2014019781A1 issued to Institut National De La Sante Et De La Recherche Medicale (Inserm), Centre National De La Recherche Scientifique, Universite Jean Monnet. Jean-Philippe Camdessanché, Jean-Christophe Antoine, Christian Moritz has patent pending to Institut National De La Sante Et De La Recherche Medicale (Inserm), Centre National De La Recherche Scientifique, Universite Jean Monnet. If there are other authors, they declare that they have no known competing financial interests or personal relationships that could have appeared to influence the work reported in this paper.

## Data Availability

Data will be made available on request.

## Glossary

**Autoantogenome**: The repertoire of autoantibody-targeted protein antigens.
**Autoantigenomics**: The systematic analysis of the autoantigenome in an omics-style.
**Overrepresentation**: Excessive representation above the expected level based on a reference group.
**Paraneoplastic**: A condition that is the consequence of a tumor.
**Protein microarray**: Slide with recombinant (full-length) proteins spotted in separated spots.
