# Supplementary material for: The antibody repertoire of autoimmune sensory neuronopathies targets pathways of the innate and adaptive immune system. An autoantigenomic approach
Source: J Transl Autoimmun. 2025 Jan 30;10:100277. doi: 10.1016/j.jtauto.2025.100277 (PMC11870273; doi:10.1016/j.jtauto.2025.100277)
Supplement: Multimedia component 1 [file mmc1.docx]

**Appendices**

**Table A.1 Numbers of proteins within Reactome pathways allocated to Immune System and numbers of proteins per pathway present on the different arrays used in this study.**

| **Reactome pathways of Immune System** | **Proteins in Pathway** | **Pathway proteins on Protoarray®** | **Pathway proteins on HuProt 3.1®** | **Pathway proteins on HuProt 4.0®** |
| --- | --- | --- | --- | --- |
| **Immune System** | 2038 | 863 | 1694 | 1786 |
| Adaptive Immune System | 821 | 335 | 628 | 678 |
| Antigen processing-Cross presentation | 100 | 43 | 87 | 96 |
| ER-Phagosome pathway | 84 | 39 | 74 | 83 |
| Class I MHC mediated antigen processing & presentation | 372 | 169 | 304 | 332 |
| Immunoregulatory interactions between a Lymphoid and a non-Lymphoid cell | 197 | 45 | 110 | 121 |
| Activation of NF-kappaB in B cells | 67 | 36 | 58 | 59 |
| Downstream TCR signaling | 103 | 53 | 88 | 89 |
| **Cytokine Signaling in Immune system** | 669 | 335 | 607 | 665 |
| Interferon alpha/beta signaling | 67 | 38 | 65 | 73 |
| Interferon gamma signaling | 91 | 44 | 87 | 93 |
| TNFR2 non-canonical NF-kB pathway | 100 | 45 | 89 | 91 |
| Signaling by Interleukins | 449 | 237 | 413 | 433 |
| Interferon Signaling | 193 | 90 | 171 | 184 |
| Interleukin-17 signaling | 71 | 49 | 65 | 67 |
| Interleukin-4 and Interleukin-13 signaling | 111 | 53 | 100 | 103 |
| Interleukin-1 family signaling | 137 | 81 | 121 | 140 |
| Interleukin-1 signaling | 101 | 64 | 89 | 104 |
| NIK-->noncanonical NF-kB signaling | 59 | 32 | 52 | 53 |
| **Innate Immune System** | 1102 | 462 | 905 | 936 |
| Dectin-1 mediated noncanonical NF-kB signaling | 60 | 33 | 53 | 54 |
| FCERI mediated NF-kB activation | 142 | 51 | 75 | 76 |
| Toll-Like Receptors Cascades | 152 | 88 | 135 | 153 |
| Cytosolic sensors of pathogen-associated DNA | 63 | 30 | 50 | 51 |
| Neutrophil degranulation | 479 | 216 | 418 | 428 |
| DDX58/IFIH1-mediated induction of interferon-alpha/beta | 77 | 39 | 66 | 73 |
| NOD1/2 Signaling Pathway | 35 | 19 | 29 | 33 |
| MAP kinase activation | 63 | 45 | 57 | 59 |
| Toll Like Receptor TLR1:TLR2 Cascade | 97 | 69 | 88 | 109 |
| MyD88:Mal cascade initiated on plasma membrane | 94 | 68 | 85 | 106 |
| Toll Like Receptor TLR6:TLR2 Cascade | 94 | 68 | 85 | 106 |
| MyD88 dependent cascade initiated on endosome | 92 | 64 | 83 | 95 |
| TRAF6 mediated induction of NFkB and MAP kinases upon TLR7/8 or 9 activation | 91 | 64 | 82 | 94 |
| MyD88 cascade initiated on plasma membrane | 84 | 61 | 75 | 90 |
| activated TAK1 mediates p38 MAPK activation | 23 | 15 | 19 | 21 |
| Activation of the AP-1 family of transcription factors | 10 | 9 | 10 | 10 |
| MyD88-independent TLR4 cascade | 97 | 67 | 89 | 101 |
| TRIF(TICAM1)-mediated TLR4 signaling | 97 | 67 | 89 | 101 |

**Table A.2. Percentage of immune system pathways covered by immunoreactivities of different patient groups.**

| **Reactome pathway of Immune System** | **% of targeted proteins on Protoarray®** | | | | | | **% of targeted proteins on HuProt 3.1®** | | | | **% of targeted proteins on HuProt 4.0®** | |
| --- | --- | --- | --- | --- | --- | --- | --- | --- | --- | --- | --- | --- |
|  | **Hu** | **SNN** | **SNN FGFR3-neg.** | **SNN FGFR3-pos.** | **ONP** | **HC** | **SNN** | **SNN anti-AGO1-negative** | **SNN anti-AGO1-positive** | **HC** | **SNN** | **HC** |
| **Immune System** | 2.5 | **31.4** | **8.1** | **30.0** | 1.6 | 1.6 | **6.6** | **5.5** | **0.6** | 2.3 | **3.1** | 0.3 |
| **Adaptive Immune System** | 3.0 | **33.1** | 7.5 | **30.7** | 1.5 | 2.4 | **7.8** | **6.1** | 0.8 | 2.7 | **3.4** | 0.3 |
| Antigen processing-Cross presentation | 2.3 | **46.5** | 9.3 | **46.5** | 0.0 | 2.3 | 9.2 | 6.9 | 0.0 | 1.1 | 4.2 | 0.0 |
| ER-Phagosome pathway | 2.6 | **46.2** | 10.3 | **46.2** | 0.0 | 2.6 | 6.8 | 5.4 | 0.0 | 1.4 | 4.8 | 0.0 |
| Class I MHC mediated antigen processing & presentation | 1.8 | **40.8** | 8.3 | **36.7** | 1.2 | 1.2 | 8.2 | 6.3 | **0.7** | 3.6 | **2.4** | 0.0 |
| Immunoregulatory interactions between a Lymphoid and a non-Lymphoid cell | 0.0 | **33.3** | 2.2 | **26.7** | 4.4 | 0.0 | 4.5 | 3.6 | 0.0 | 0.9 | 6.6 | 0.8 |
| Activation of NF-kappaB in B cells | 5.6 | **38.9** | 13.9 | **38.9** | 0.0 | 5.6 | 8.6 | 5.2 | 0.0 | 1.7 | 5.1 | 0.0 |
| Downstream TCR signaling | 3.8 | **35.8** | 11.3 | **35.8** | 0.0 | 5.7 | 6.8 | 4.5 | 0.0 | 1.1 | 4.5 | 0.0 |
| **Cytokine Signaling in Immune system** | 2.1 | **33.7** | **10.1** | **33.4** | 1.5 | 2.4 | **6.4** | **5.3** | 1.0 | 2.1 | **3.5** | 0.5 |
| Interferon alpha/beta signaling | 2.6 | **39.5** | 7.9 | **39.5** | 2.6 | 0.0 | 0.0 | 0.0 | 0.0 | 3.1 | 1.4 | 0.0 |
| Interferon gamma signaling | 2.3 | **34.1** | 2.3 | **29.5** | 4.5 | 2.3 | 6.9 | 4.6 | 1.1 | 2.3 | 2.2 | 0.0 |
| TNFR2 non-canonical NF-kB pathway | 2.2 | **37.8** | 13.3 | **33.3** | 0.0 | 6.7 | 5.6 | 2.2 | 1.1 | 1.1 | 4.4 | 0.0 |
| Signaling by Interleukins | 3.0 | **34.6** | **12.2** | **34.2** | 1.3 | 2.5 | **7.0** | **5.8** | 0.7 | 1.9 | **4.2** | 0.7 |
| Interferon Signaling | 1.1 | **33.3** | 4.4 | **32.2** | 2.2 | 2.2 | 5.8 | 3.5 | 1.2 | 3.5 | 1.6 | 0.0 |
| Interleukin-17 signaling | 4.1 | **38.8** | **20.4** | **44.9** | 0.0 | 0.0 | 10.8 | 7.7 | 0.0 | 0.0 | 4.5 | 0.0 |
| Interleukin-4 and Interleukin-13 signaling | 1.9 | **32.1** | 7.5 | **39.6** | 0.0 | 5.7 | 7.0 | 5.0 | 2.0 | 3.0 | 3.9 | 1.9 |
| Interleukin-1 family signaling | 4.9 | **38.3** | 14.8 | **38.3** | 1.2 | 2.5 | 7.4 | 6.6 | 0.0 | 2.5 | 4.3 | 0.0 |
| Interleukin-1 signaling | 4.7 | **40.6** | 14.1 | **35.9** | 1.6 | 3.1 | 7.9 | 5.6 | 0.0 | 3.4 | 2.9 | 0.0 |
| NIK-->noncanonical NF-kB signaling | 3.1 | **46.9** | 18.8 | **40.6** | 0.0 | 3.1 | 9.6 | 3.8 | 1.9 | 1.9 | 5.7 | 0.0 |
| **Innate Immune System** | 3.7 | **30.1** | **9.7** | **29.0** | 1.1 | 1.3 | **7.5** | **6.1** | 0.4 | 1.7 | **2.9** | 0.2 |
| Dectin-1 mediated noncanonical NF-kB signaling | 3.0 | **45.5** | 18.2 | **39.4** | 0.0 | 6.1 | 9.4 | 3.8 | 1.9 | 1.9 | 5.6 | 0.0 |
| FCERI mediated NF-kB activation | 3.9 | **35.3** | 11.8 | **31.4** | 2.0 | 5.9 | 9.3 | 6.7 | 0.0 | 1.3 | 5.3 | 1.3 |
| Toll-Like Receptor Cascades | 6.8 | **31.8** | **14.8** | **35.2** | 1.1 | 1.1 | **12.6** | **8.9** | **0.7** | 1.5 | 2.6 | 0.0 |
| Cytosolic sensors of pathogen-associated DNA | 3.3 | 23.3 | 6.7 | **30.0** | 0.0 | 3.3 | 6.0 | 2.0 | 0.0 | 2.0 | 2.0 | 0.0 |
| Neutrophil degranulation | 2.8 | **27.8** | **9.3** | **24.1** | 1.4 | 0.0 | **5.7** | **4.8** | 0.5 | 1.7 | **2.8** | 0.0 |
| DDX58/IFIH1-mediated induction of interferon-alpha/beta | 0.0 | 23.1 | 2.6 | **30.8** | 2.6 | 2.6 | 3.0 | 1.5 | 0.0 | 1.5 | 2.7 | 0.0 |
| NOD1/2 Signaling Pathway | 5.3 | 26.3 | 15.8 | **42.1** | 0.0 | 0.0 | 6.9 | 3.4 | 0.0 | 0.0 | 9.1 | 0.0 |
| MAP kinase activation | 4.4 | **40.0** | **22.2** | **46.7** | 0.0 | 0.0 | 10.5 | 7.0 | 0.0 | 0.0 | 5.1 | 0.0 |
| Toll Like Receptor TLR1:TLR2 Cascade | 5.8 | **37.7** | **17.4** | **39.1** | 0.0 | 1.4 | **13.6** | 10.2 | 0.0 | 1.1 | 2.8 | 0.0 |
| MyD88:Mal cascade initiated on plasma membrane | 5.9 | **38.2** | **17.6** | **39.7** | 0.0 | 1.5 | **12.9** | 10.6 | 0.0 | 1.2 | 2.8 | 0.0 |
| Toll Like Receptor TLR6:TLR2 Cascade | 5.9 | **38.2** | **17.6** | **39.7** | 0.0 | 1.5 | **12.9** | 10.6 | 0.0 | 1.2 | 2.8 | 0.0 |
| MyD88 dependent cascade initiated on endosome | 4.7 | **37.5** | **18.8** | **39.1** | 0.0 | 1.6 | 10.8 | 8.4 | 0.0 | 1.2 | 3.2 | 0.0 |
| TRAF6 mediated induction of NFkB and MAP kinases upon TLR7/8 or 9 activation | 4.7 | **37.5** | **18.8** | **39.1** | 0.0 | 1.6 | 11.0 | 8.5 | 0.0 | 1.2 | 3.2 | 0.0 |
| MyD88 cascade initiated on plasma membrane | 4.9 | **39.3** | **19.7** | **41.0** | 0.0 | 1.6 | 10.7 | 8.0 | 0.0 | 1.3 | 3.3 | 0.0 |
| activated TAK1 mediates p38 MAPK activation | 6.7 | 40.0 | 13.3 | **46.7** | 0.0 | 0.0 | 10.5 | 5.3 | 0.0 | 0.0 | 4.8 | 0.0 |
| Activation of the AP-1 family of transcription factors | 0.0 | 33.3 | 22.2 | **88.9** | 0.0 | 0.0 | 10.0 | 10.0 | 0.0 | 0.0 | 20.0 | 0.0 |
| MyD88-independent TLR4 cascade | 3.0 | **31.3** | 14.9 | **37.3** | 1.5 | 1.5 | 9.0 | 6.7 | 0.0 | 1.1 | 3.0 | 0.0 |
| TRIF(TICAM1)-mediated TLR4 signaling | 3.0 | **31.3** | 14.9 | **37.3** | 1.5 | 1.5 | 9.0 | 6.7 | 0.0 | 1.1 | 3.0 | 0.0 |

The proportion of pathway coverage is represented as a both values and shades of color ranging from light blue (low percentage) to red (high percentage). In addition. these percentages for each pathway in each patient group are compared to that of blood donors (HC) for each protein array used. If the difference is statistically significant after Benjamini-Hochberg correction (p≤0.05), the percentage value is in bold.
